# The Evolving Stethoscope: Insights Derived from Studying Phonocardiography in Trainees

**DOI:** 10.3390/s24165333

**Published:** 2024-08-17

**Authors:** Matthew A. Nazari, Jaeil Ahn, Richard Collier, Joby Jacob, Halen Heussner, Tara Doucet-O’Hare, Karel Pacak, Venkatesh Raman, Erin Farrish

**Affiliations:** 1 Department of Internal Medicine and Pediatrics, MedStar Georgetown University Hospital, Washington, DC 20007, USA; 2 Department of Biostatistics, Bioinformatics, and Biomathematics, MedStar Georgetown University Hospital, Washington, DC 20007, USA; 3 Boston Children’s Hospital, Department of Pediatric Cardiology, Boston, MA 02115, USA; 4 Beth Israel Deaconess Medical Center, Department of Internal Medicine, Boston, MA 02215, USA; 5 Arizona College of Osteopathic Medicine, Midwestern University, Glendale, AZ 85308, USA; 6 National Cancer Institute, Center for Cancer Research, Neuro-Oncology Branch, Bethesda, MD 20892, USA; tara.doucet-o’; 7 Section on Medical Neuroendocrinology, Eunice Kennedy Shriver National Institute of Child Health and Human Development, National Institutes of Health, Bethesda, MD 20892, USA; 8 Veterans Affairs Medical Center, Division of Cardiology, Washington, DC 20422, USA

**Keywords:** phonocardiography, auscultation, educational technology, digital stethoscope, testing, evaluation, medical education, teaching, learning, telemedicine

## Abstract

Phonocardiography (PCG) is used as an adjunct to teach cardiac auscultation and is now a function of PCG-capable stethoscopes (PCS). To evaluate the efficacy of PCG and PCS, the authors investigated the impact of providing PCG data and PCSs on how frequently murmurs, rubs, and gallops (MRGs) were correctly identified by third-year medical students. Following their internal medicine rotation, third-year medical students from the Georgetown University School of Medicine completed a standardized auscultation assessment. Sound files of 10 different MRGs with a corresponding clinical vignette and physical exam location were provided with and without PCG (with interchangeable question stems) as 10 paired questions (20 total questions). Some (32) students also received a PCS to use during their rotation. Discrimination/difficulty indexes, comparative chi-squared, and McNemar test *p*-values were calculated. The addition of phonocardiograms to audio data was associated with more frequent identification of mitral stenosis, S4, and cardiac friction rub, but less frequent identification of ventricular septal defect, S3, and tricuspid regurgitation. Students with a PCS had a higher frequency of identifying a cardiac friction rub. PCG may improve the identification of low-frequency, usually diastolic, heart sounds but appears to worsen or have little effect on the identification of higher-frequency, often systolic, heart sounds. As digital and phonocardiography-capable stethoscopes become more prevalent, insights regarding their strengths and weaknesses may be incorporated into medical school curricula, bedside rounds (to enhance teaching and diagnosis), and telemedicine/tele-auscultation efforts.

## 1. Introduction

Cardiac auscultation is a time-honored bedside clinical skill and when performed by a skilled examiner can be an effective tool for identifying valvular heart disease (sensitivity: 70%; specificity: 98% [1] for valvular disease; sometimes up to 95–100% [2] sensitivity/specificity with maneuvers and dynamic auscultation). However, recent data indicate suboptimal proficiency in cardiac auscultation among trainees (20–54% [3,4,5] diagnostic accuracy) who show little improvement after an additional year of training (~10% [6] in one study) and attain a level of proficiency only slightly better than that of third-year medical students by the end of residency [4]. Thus, the initial training in cardiac auscultation in third-year medical students may define a pivotal moment for shaping proficiency and accuracy in correctly identifying heart sounds.

Recently, there has been less of an emphasis on structured training in cardiac auscultation (about 25% [7] of internal medicine and family practice residencies and 37% of cardiovascular disease fellowship programs) while teaching time at the bedside has become infrequent (17%) [8], even at academic institutions (like Brigham and Women’s Hospital). Some authorities have also attributed the availability of other technologies (e.g., echocardiography), in part, to the declining accuracy in recognizing cardiac events [9] while others have even deemed the stethoscope an “outmoded” instrument [4].

In response, various interventions have focused on improving accuracy in cardiac auscultation in trainees using structured teaching (about 60% improvement) [10], simulated heart sounds (about 20%) [5], multimedia technology-based learning tools (about 20%) [11], and repetition (about 70%) [12]. Advances in technology have also led to a resurgence of interest in phonocardiography (PCG), a graphical display of heart sounds throughout the cardiac cycle.

W. Proctor Harvey became an authority in using PCG to teach the identification of heart sounds to trainees during his tenure at Georgetown University Hospital from 1950 until the late 1990s [13,14,15,16]. While auscultation collects mechanical vibrations over the body’s surface, comprising the full spectrum of sound frequencies (20–20,000 Hz), the human ear struggles to perceive lower-frequency vibrations (<100 Hz) with infrasonic vibrations (<20 Hz) being imperceptible [13,17]. PCG, however, provides a graphic display transcending these limitations and enhancing the identification of low-frequency vibrations [13,17]. Consequently, PCG was central to the studies conducted in the 1980s and 1990s investigating the origin of the low-frequency S3 gallop (15–60 Hz) and has been considered an integral tool for gallop (e.g., S3/S4) detection [18,19]. Unfortunately, at that time, PCG was time-consuming, cumbersome, and required immobile bulky equipment, obviating its practical use at the bedside [13,20]. As a result, by the late 1990s, PCG was largely discarded in the United States [13,20].

In a pioneering study published by Morton E. Tavel and colleagues in 1994, a handheld electronic device that connected to a stethoscope was described that provided a graphical display of heart sounds (in essence, a PCG) [20]. It was found by two expert cardiologists—performing simultaneous auscultation and an analysis of the graphic display—that auscultation missed about 15% of murmurs, rubs, or gallops (MRGs) and that these sounds were largely low-frequency and evinced by the graphic display (or PCG). However, the graphic display tended to obscure high-frequency, low-intensity (<grade 2) murmurs (e.g., mitral and aortic regurgitation) and sounds with rapid split transients (e.g., a split S2 or an S3 in close apposition to the S2) [20]. The availability of digital and PCG-capable stethoscopes (PCSs) in modern times allows for further investigation into the utility of PCG as a learning tool in trainees but also in artificial intelligence (AI)-assisted auscultation given that all AI pipelines rely upon PCG for subsequent analysis [21,22,23,24,25].

Nevertheless, despite advances, AI is limited by interference from pulmonary and gastrointestinal sounds [20], has a range of accuracies across PCS models (88–96% accurate) [24,26], and has only been useful in making binary decisions (e.g., normal versus abnormal; sensitivity: 97%; specificity: 89%) [26]. These constraints have led certain experts to deem AI assistance in auscultation a support tool only, not a standalone system [27]. Regardless, PCG generated by a digital stethoscope, whether subsequently analyzed by AI or not, has potentially significant applications in aiding telemedicine evaluation and in tele-auscultation [28,29].

In light of these studies, it seems that PCG will become more available with the development of digital stethoscopes, may aid in identifying low-frequency heart sounds, and may increasingly be incorporated into telemedicine evaluations while possibly obscuring high-frequency, low-intensity heart sounds (recall the Tavel study) [20]. It is, thus, the very next generation of medical providers (e.g., third-year medical students) that are most poised to use these technologies and, therefore, are a particularly important population to study. To date, no prior studies have been performed evaluating the impact of PCG/PCS on the identification of different MRGs in third-year medical students and further, it is unclear whether such technologies will provide a clear superiority over the traditional stethoscopes or be preferred moving forward by third-year medical students. Recognizing these current gaps in understanding, we devised the present study to investigate the impact of PCGs and PCSs on the identification of all MRGs in those most immediately expected to enter clinical practice—third-year medical students.

## 2. Materials and Methods

Third-year medical students (*n* = 196) undergoing their internal medicine rotation (between August 2020 and June 2021) at the Georgetown University School of Medicine were recruited into the study. A survey was sent to the students before the start of their third-year internal medicine rotation soliciting interest in PCS use. Core didactics included a one-hour recorded session on cardiac auscultation with an introduction to PCG and examples of abnormal findings. Fifty-four students agreed to be a part of the study (*n* = 54/196, 27.6%). We had six PCSs (seven toward the end of the study) and assigned these to the study participants using a random name generator. Ultimately, 32 students (*n* = 32/54, 59.3%) received a PCS throughout the study.

A subsequent survey was sent to the participants within the arm who agreed to be a part of the study (*n* = 54) requesting demographic data (displayed in Table 1). Three participants within the PCS group and three within the group without a PCS did not provide demographic data.

The EKO Core Digital Stethoscope^®^, a PCS, was utilized. The model provides up to 40× amplification, includes a diaphragm and bell, and allows for real-time visualization of PCGs with the ability to record heart sounds.

Sound files of 10 MRGs (5 valvopathies, 2 gallops, 1 cardiac friction rub, 1 ventricular septal defect, and 1 split S2) with a corresponding clinical vignette and cardiac exam location were provided with and without PCG (with interchangeable question stems) as 10 paired questions (20 total questions). In this way, the same recording of a heart sound was provided both with and without a PCG (forming a dyad), so each question of the pair had a different clinical vignette. These 20 questions were provided with 8 additional clinical questions forming a 28-question practical exam. These additional questions focused on pulmonary, radiographic, and electrocardiogram findings.

The demographic variables and test scores for the two participant groups or those provided with a PCS (*n* = 32) and those without a PCS (*n* = 22) are summarized in Table 1. For the two group comparisons, Pearson’s chi-squared/the binomial exact test was used for categorical variables, and two-sample *t*-tests were used for continuous variables. To further explore the utility of PCG, as incorporated for specific questions, we used a 2-parameter item-response logistic model to characterize the question difficulty (intercept) and question discrimination (slope) indexes. The percent (%) difference in the correct answer was calculated for (1) the total cohort (*n* = 196), (2) the participants with a PCS (*n* = 32), (3) the participants without a PCS (*n* = 22), and (4) the total cohort without a PCS (*n* = 164). We used the McNemar test to assess if there were any differences in the correct answer proportions with and without PCG for each MRG type question with different difficulty and discrimination levels (Table 2). The findings were determined to be statistically significant if the two-sided *p*-value was <0.05; multiplicity correction was not employed.

**Figure 1:**
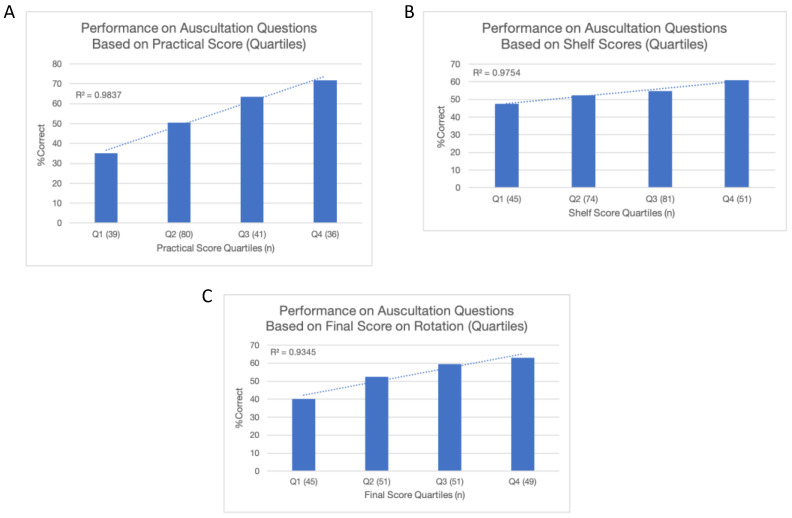
Correlates of performance on cardiac auscultation questions. (**A**) Displays the relation of performance on auscultation questions to practical exam score, (**B**) shelf exam score, and (**C**) final score on the internal medicine rotation.

Performance on the practical exam (all 28 questions on the survey tool), on the internal medicine shelf exam, and in the overall class (a weighted average of the practical score, shelf score, clinical evaluations, and other practical tests, collectively, the final score on the rotation) was recorded. Performance metrics (practical, shelf exam, and final score on the rotation) were divided into interquartile ranges (Q1–4) and correlated with performance on the 20 auscultation questions along with a line of best fit and coefficient of determination (*R*^2^) calculated (Figure 1). All of the statistical analysis was carried out using the *R* software version 3.4 (R Foundation for Statistical Computing, Vienna, Austria).

## 3. Results

### 3.1. Demographic Information

Within the group of study participants, there was no statistically significant difference regarding age (*p* = 0.74), gender (*p* = 0.14), or ethnicity (*p* = 0.69) between those who received a PCS and those who did not (Table 1). Additional demographic information was not obtained in the total cohort that did not participate in the study.

### 3.2. Question Difficulty and Discrimination

Amongst the 20 questions, individual discrimination and difficulty indexes were calculated (Appendix A). The questions with a difficulty index of ≤0.50 were deemed hard, >0.5 and <0.85 moderate, and ≥0.85 easy. A question with a discrimination index of ≥0.2 was considered permissible and, therefore, adequate for interpretation, while a question with a discrimination index < 0.2 should be interpreted with caution (as in Table 2); these included tricuspid regurgitation, S3, aortic stenosis, and mitral stenosis in this study. There was no difference in difficulty (*p* = 0.60) or discrimination (*p* = 0.91) between the 10 questions in which a PCG was provided and the 10 questions in which a PCG was not provided.

### 3.3. PCG and PCS

The addition of PCG to audio data and the use of PCS was associated with a higher frequency of identification (*p* < 0.001) of mitral stenosis (PCG only), an S4 gallop (PCG only), and a cardiac friction rub (PCG and PCS) and a lower rate of identification (*p* ≤ 0.001) of ventricular septal defect (PCG and PCS), an S3 gallop (PCG and PCS), and tricuspid regurgitation (PCG and PCS) [Table 2].

### 3.4. Correlates

Performance on the auscultation questions correlated positively with performance on the practical exam (*R*^2^ = 0.98), shelf exam (*R*^2^ = 0.98), and final score on the rotation (*R*^2^ = 0.93) [Figure 1].

## 4. Discussion

The effect of PCG on identifying heart sounds was mixed in this study. As suggested by prior studies, the heart sounds more frequently identified with the provision of PCG were often diastolic and low-frequency (e.g., 25–125 Hz, including S4 at 15–45 Hz and mitral stenosis at 45–90 Hz) or had a low-frequency component (e.g., cardiac friction rub with a low-frequency component of 100 Hz). The heart sounds that were systolic (excepting aortic regurgitation) and high-frequency (e.g., >300 Hz; including aortic regurgitation at 60–380 Hz, split S2 at 50–400 Hz, mitral regurgitation at 60–400 Hz, and aortic stenosis at 100–400 Hz) were not associated with more frequent identification when PCG was provided [30]. The heart sounds that were associated with less frequent identification with PCG were often systolic and moderate (125–300 Hz) or high-frequency (including ventricular septal defect at 50–180 Hz and tricuspid regurgitation at 90–400 Hz) with the notable exception of S3, a diastolic, low-frequency (15–60 Hz) heart sound [30].

As mentioned above, infrasonic (<20 Hz) and low-frequency (<100 Hz) heart sounds are challenging for the human ear to perceive, are often diastolic, and therefore, missed by trainees (in up to 60% of trainees) and experienced providers alike, suggesting that they are inherently challenging to identify [6,17,30,31]. While PCG makes the identification of these low-frequency, often diastolic heart sounds easier to identify, the high-frequency, low-intensity, often systolic heart sounds or those with rapid split transients (like an S3) are usually missed, suggesting that auscultation and PCG have strengths that address their corresponding deficits [20]. This was also apparent in our study. Further, the S3 was more often missed with PCG, as demonstrated in other studies in which S3 has been less often identified by learners (correct identification 6%) and has improved little, even when PCG is added (2% improvement in first-year medical students) [6,25]. Unfortunately, the PCS group only demonstrated a statistically significant improvement in the identification of the cardiac friction rub, which may have been because of a lower detectable effect size or because we were not able to know just how much students provided with a PCS actually used it. Thus, findings from the PCG group may still be applicable to PCSs but further investigation is needed.

### 4.1. Study Considerations: Strengths, Limitations, and Improvements

This study assesses the use of PCG/PCS in the identification of different MRGs using a large sample size (*n* = 196) of third-year medical students. Exposure to PCG/PCS was largely limited in the curriculum preceding the third year of clinical rotations and thus, a standard orientation to auscultation as well as PCG with abnormal findings was provided to all the students to address possible bias from this limited/heterogenous prior exposure.

Data on how frequently a PCS was used and the willingness of third-year medical students to embrace this technology was not collected, which may have allowed for greater insight into the observed associations. Within a dyad, one question (with PCG for example) may have had a different difficulty compared to another question (without PCG) which may have influenced participant accuracy (Appendix A). Not all of the questions were highly discriminant, limiting the interpretation of the results for certain heart sounds. In addition, a control group of individuals with more experience (for example, attending cardiologists) would have provided a valuable comparator, especially with regard to assessing question characteristics. Finally, a multicenter, multiregional study (as opposed to this single institution study) would have allowed for greater generalizability.

### 4.2. Implications in Practice and Medical Education

The original stethoscope, invented in 1816, comprised a wooden tube placed on the chest and with serial iterations, tubing, ear pieces, and a bell pickup device (to preserve low-frequency sounds) was added, sequentially enhancing the stethoscope’s utility [20,32]. Over time, medicine has become more busy, sometimes rushed, and noisy, making careful auscultation progressively more challenging while placing a greater emphasis on more objective diagnostic evaluations with permanent records (echocardiography for example) [21]. The development of digitalized diagnostic tools such as PCS may be able to address some of these challenges encountered in the modern era of medicine.

A PCS can amplify low-frequency sounds (up to 40× in the low-frequency band of 125 Hz), provide an objective permanent record (a PCG), and soon [with AI assistance] provide binary, yet helpful information (normal versus abnormal or systolic versus diastolic)—perhaps welcomed innovations in light of more recent constraints and sensibilities [20,22]. Imagine a stethoscope that directs the provider’s attention to an abnormal diastolic sound that with amplification and further inspection of a corresponding PCG is more correctly identified all in less than a minute. Such a scenario may soon be possible and may be the commensurate innovation needed for the stethoscope of the future to meet the new demands of clinical practice defining the stethoscope’s “second act” in re-establishing its dominance and utility at the bedside. Furthermore, it is the imminent clinician-to-be, the third-year medical student, and the vanguard of future generations of providers that must be the subject of study if we are to understand and support the advancement of physical examination and diagnosis.

Finally, advancements in auscultation and phonocardiography may extend the reach of providing medical care to more patients in rural and underserved areas, as these patients may have challenges in traveling to healthcare sites. This increasing prevalence of telemedicine may provide the opportunity for tele-auscultation, allowing more equitable delivery of healthcare to rural and underserved areas. In addition, trainees and preceptors can secure a more permanent record of such heart sounds and review these tele-auscultated sounds together, extending teaching to the virtual bedside.

## 5. Conclusions

Looking forward, and in the words of some distinguished clinicians and educators, the stethoscope is not dead, it is in fact “healthier than ever” [33]. Even more so, it may be evolving when one considers the possibilities of integrating newer advancements like PCG.

All medical students at the Georgetown University School of Medicine, until this day, receive a three-headed stethoscope, the Harvey DLX Triple Head^®^, with a conventional diaphragm, bell, and corrugated diaphragm that, among other functions, allows one to distinguish heart sounds of different frequencies. In the spirit of medical education and innovation—embodied by Dr. Proctor Harvey—the “next-generation” stethoscope may be a PCS even more capable of enhancing lower-frequency heart sounds.

## Figures and Tables

**Table 1:** Demographic information and test scores (*n* = 196).

*n* (%) or Mean (SD)	With PCS (*n* = 32)	Without PCS (*n* = 22)	Cohort without PCS (*n* = 164)
**Age (y)** *[p = 0.74]*	*n* = 29 ^a^	*n* = 19 ^a^	N/A
20–24	4 (13.8%)	2 (10.5%)	
25–29	20 (69.0%)	15 (78.9%)	
30–34	5 (17.2%)	2 (10.5%)	
**Gender (F)** *[p = 0.14]*	17 (58.6%)	7 (36.8%)	
**Ethnicity** *[p = 0.69]*			
Asian	7 (24.1%)	6 (31.6%)	
Black or African American	2 (6.9%)	1 (5.3%)	
Hispanic or Latino	1 (3.4%)	2 (10.5%)	
White	19 (65.5%)	10 (52.6%)	
**Test Scores**	*n* = 32	*n* = 22	*n* = 164
Practical Score	60.6 (12)	61.5 (11) ^b^	61.7 (12) ^b^
Shelf Score	72.5 (8)	74.1 (8) ^b^	74.2 (8) ^b^
Final Score	319.5 (37)	326.3 (30) ^b^	324.6 (39) ^b^

Abbreviations: SD: standard deviation; PCS: phonocardiography capable stethoscope. ^a^ Three participants in the group with PCS and three within the group without PCS did not provide demographic information. ^b^ No statistically significant difference compared to those with a PCS.

**Table 2:** Performance of third-year Georgetown University medical students on auscultation questions with and without PCG and PCS (*n* = 32/196 with PCS).

*Murmur* ^a^	Total Cohort (*n* = 196)				With PCS	Without PCS	Total Cohort without PCS	Effect on Identification
	% Correct with PCG	% Correct without PCG	% Difference ^b^	McNemar χ2 (*p*-Value)	% Difference (*p*-Value)			
*Aortic Regurgitation*	47.7	54.4	−6.7	2.6 (0.11)	−9.4 (0.32)	0 (1.00)	−6.1 (0.18)	No difference
*S4*	86.7	68.7	+18.0	22.3 (<0.001)	+9.4 (0.37)	+22.7 (0.06)	+19.5 (<0.001)	More frequent with PCG
*Cardiac Friction Rub*	79.0	42.6	+36.2	58.0 (<0.001)	+40.6 (<0.001)	+45.5 (0.004)	+35.4 (<0.001)	More frequent with PCG and PCS
*Split S2*	65.1	66.7	−1.6	0.14 (0.71)	+6.3 (0.53)	−13.6 (0.26)	−3.0 (0.49)	No difference
*Mitral Regurgitation*	56.4	59.0	−2.6	0.32 (0.57)	+12.5 (0.25)	−18.2 (0.16)	−3.0 (0.57)	No difference
*Ventricular Septal Defect*	14.4	51.3	−36.9	61.7 (<0.001)	−46.9 (<0.001)	−27.3 (0.01)	−34.8 (<0.001)	Less frequent with PCG and PCS
*Tricuspid Regurgitation*	33.3	75.4	−42.1	61.1 (<0.001)	−53.1 (0.001)	−27.3 (0.06)	−39.6 (<0.001)	Less frequent with PCG and PCS ^c^
*S3*	7.7	60.0	−52.3	96.3 (<0.001)	−56.3 (<0.001)	−50.0 (0.002)	−51.2 (<0.001)	Less frequent with PCG and PCS ^c^
*Aortic Stenosis*	95.4	96.9	−1.5	1.1 (0.29)	−6.3 (0.32)	−4.5 (0.32)	−1.2 (0.53)	No difference ^c^
*Mitral Stenosis*	18.0	5.1	+12.9	17.0 (<0.001)	+6.3 (0.41)	+13.6 (0.08)	+14.0 (<0.001)	More frequent with PCG ^c^

Abbreviations: PCG: phonocardiogram; PCS: phonocardiography-capable stethoscope. ^a^ From a two-parameter, item-response, logistic model questions are sorted by (A) discrimination index (most discriminant toward the top and least discriminant toward the bottom) and (B) by difficulty index: hard questions, moderately difficult questions, and easy questions. ^b^ Displays the difference in percent (%) correct within each dyad (with and without PCG for the same heart sound) for the total group of participants (left), and % difference (right) for participants that received a PCS (*n* = 32), participants that did not receive a PCS, (*n* = 22), and the total cohort without a PCS (*n* = 196). ^c^ Interpret results with caution.

## Data Availability

The original contributions presented in the study are included in the article/Supplementary Digital Appendix, further inquiries can be directed to the corresponding author.

## Supplementary Materials

The following supporting information can be downloaded at: https://www.mdpi.com/article/10.3390/s24165333/s1, Supplementary Digital Appendix: Displays the anonymized raw data collected (Table S1), and subsequent statistical analyses (Tables S2–S5).

## Appendix A. Discrimination and Difficulty Index on Cardiac Auscultation Questions

**Murmur**	**Without PCG**		**With PCG**	
	**Discrimination Index**	**Difficulty Index**	**Discrimination Index**	**Difficulty Index**
Split S2	0.22	0.62	0.42	0.63
MS	0.02	0.03	0.09	0.19
VSD	0.23	0.45	0.15	0.15
CFR	0.28	0.42	0.26	0.76
AS	0.09	0.94	0.06	0.95
S3	0.18	0.58	0.03	0.07
TR	0.14	0.67	0.11	0.23
AR	0.37	0.52	0.35	0.51
S4	0.35	0.67	0.22	0.85
MR	0.17	0.60	0.29	0.51
Abbreviations: AR: aortic regurgitation; AS: aortic stenosis; CFR: cardiac friction rub; MR: mitral regurgitation; MS: mitral stenosis; TR: tricuspid regurgitation; VSD: ventricular septal defect.				
